# Changes in the prehospital emergency technician's resilience during the COVID‐19 pandemic: A web‐based cross‐sectional study

**DOI:** 10.1002/hsr2.1223

**Published:** 2023-04-19

**Authors:** Abbasali Ebrahimian, Asghar Keshavarz‐Tork, Yousof Akbari‐Shahrestanaki, Maedeh Tourdeh, Ali Fakhr‐Movahedi

**Affiliations:** ^1^ Health in Emergencies and Disasters Group, Faculty of Paramedical Qom University of Medical Sciences Qom Iran; ^2^ Nursing Care Research Center Semnan University of Medical Sciences Semnan Iran; ^3^ Nursing School Semnan University of Medical Sciences Semnan Iran; ^4^ Department of Prehospital Emergency Medical Care, School of Paramedical Sciences Qazvin University of Medical Sciences Qazvin Iran; ^5^ Anesthesia Department, Faculty of Paramedical Qom University of Medical Sciences Qom Iran

**Keywords:** COVID‐19, prehospital, resilience

## Abstract

**Background and Aims:**

Resilience is a process that enables people to control the stressors of their lives. During the COVID‐19 crisis, work stress increased among prehospital emergency technicians. So, it was possible to reduce their resilience. This study aimed to investigate the changes in the prehospital emergency technicians' resilience during the pandemic of COVID‐19.

**Methods:**

A cross‐sectional study was conducted at the prehospital emergency department in Qazvin province. For 6 months, 234 emergency technicians participated in this study. Data collection tools included a demographic questionnaire and the emergency medical services resilience scale (EMSRS).

**Results:**

The Friedman test indicated no significant difference between the mean scores of EMS employees' resilience during 6 months (*p* > 0.05). However, the correlation matrix between the scores of EMSRS during 6 months indicated that the resilience scores of EMS employees were positively correlated during the study (*p* < 0.01).

**Conclusions:**

The EMS technicians' resilience was almost constant and moderate during the 6 months of care for patients with COVID‐19 and their transfer to the hospital, indicating that the COVID‐19 crisis could suppress the emergency medical technicians ability to increase resilience.

## 1 INTRODUCTION

Resilience is a process that enables people to control stressors in their lives,^1^ but it is important to understand how resilience is built.^2^ Emergency medical services (EMS) is a system in which resilience is crucial. The EMS employees are under pressure and experience much job stress due to frequent exposure to patients' pain, suffering, and casualties.^3^ These stressors can affect their resilience. The reduction of resilience affects the EMS employees' body and mind and brings about job burnout and some mental disorders such as depression, stress, anxiety, and low quality of their work and reduces the quality of EMS services.^4^, ^5^ Bentley et al. found that 6.8% of emergency medical personnel suffered from depression, 6% from nervousness, and 5.9% from stress. Some of the prehospital emergency personnel, who had more than 16 years of work experience, showed higher levels of anxiety and depression than other personnel.^6^ Bennett et al. examined mental health problems among UK prehospital emergency staff. They found that prehospital emergency personnel had high anxiety, depression, and posttraumatic stress disorder.^7^

The prehospital emergency system was a system that faced a significant increase in workload during the COVID‐19 pandemic.^8^, ^9^ Meese et al. found that COVID‐19 caused high psychological distress in 82% of nurses, clinical support staff, and advanced care providers, which affected the degree of resilience in the staff.^10^

Increasing the number of prehospital emergency contacts, increasing the number of missions, the inevitability of personnel using personal protective equipment (PPE), increasing the number of work shifts, worrying about themselves and their family members to get the COVID‐19, seeing critically ill patients, seeing patients' death, and finding out about the illness of some colleagues and their families made working in a prehospital emergency very difficult, and the employees were physically and mentally stressed.^8^, ^10^, ^11^, ^12^, ^13^ Furthermore, it was not possible to control the disease, and the EMS staff were affected by the disease for a long time.^14^ Studies have indicated that coronaviruses, like Ebola and Zika, could affect the resilience of health systems and jeopardize their ability to respond and return.^15^, ^16^ Therefore, it was likely that the EMS staff's resilience and performance would be affected over time; hence, a study was conducted to investigate changes in the resilience of prehospital emergency staff during the pandemic of COVID‐19.

## 2 METHODS

### 2.1 Study design and participants

A descriptive cross‐sectional analytical study lasted from April 8 to September 21, 2020. This study was double blinded. The participants and the sampler did not know and see each other. In addition, the participants were blinded for the statistical analyst. Prehospital emergency staff who met the inclusion criteria participated in the study as research samples.

#### 2.1.1 Inclusion criteria

1. Working in the prehospital emergency department as an Emergency medical technicians (EMTs).
2. Participation in the transfer of patients with Covid‐19.
3. The EMTs does not have previously known mental and physical disorders.

#### 2.1.2 Exclusion criteria

1. Unwillingness to continue participating in this research.
2. Suffer from physical injuries (such as Covid‐19) or mental disorders during the study period.

The sampling method used in this study was a census. In Iran, all prehospital emergency workers study at Iranian universities based on a common educational curriculum. Therefore, in terms of educational level, almost all were the same. In addition, the Iran prehospital emergency organization is responsible for managing this system, and the working and management conditions are almost the same throughout Iran. Moreover, during the Covid‐19 pandemic in Iran, all prehospital emergency workers were working according to the same instructions. Therefore, the participants in this study could largely represent prehospital emergency personnel in Iran.

### 2.2 Setting

The research was conducted in a prehospital emergency department in Qazvin Province. Qazvin Province is 1 of the 31 provinces of Iran and is located in the northwestern part of the country. The prehospital emergency system was launched in Iran in 1974. After Canada, Australia, and America, Iran was the fourth country to launch a prehospital emergency system. In 2020, Iran's prehospital emergency system had 19,800 personnel, 2190 stations, 4500 ground ambulances, 500 motorlances, 52 ambulance buses, and 42 rescue helicopters.

The Qazvin Province covers 1% of Iran. The province covers an area of approximately 15,820 square kilometers. The population of this province in 2020 was approximately 1,326,400 people. This study was conducted in 2020. This study was conducted in 2020. The prehospital emergency of Qazvin province had 351 personnel, 48 bases, 61 ground ambulances, 1 ambulance bus, and 1 rescue helicopter. The annual Qazvin EMS dispatching was 60,000.

### 2.3 Data measurement and variables

Data collection tools included a demographic questionnaire and the emergency medical services resilience scale (EMSRS).^17^

The demographic questionnaire assessed variables such as age, marital status, body mass index (BMI), the number of children, work experience, the number of shifts over the past month, the number of night shifts over the past month, work experience in EMS, and the number of critical cases of COVID‐19 patients over the past month.

EMSRS was a 31‐item resilience scale with six factors: job motivation, communication challenges, social support, remaining calm, self‐management or self‐care, and consequences of stress. Factor 1 contained 13 questions about job motivation (questions 1, 2, 3, 4, 5, 11, 12, 13, 14, 15, 16, 17, and 18). Factor 2 contained five information questions about self‐management or self‐care (questions 6, 7, 8, 9, and 26). Factor 3 contained five questions about the remaining calm (questions 10, 22, 23, 27, and 28). Factor 4 contained three questions about communication challenges (questions 19, 20, and 21). Factor 5 contained two questions about social support (questions 24 and 25). Factor 6 contained three questions about the consequences of stress (questions 29, 30, and 31). A 5‐point Likert scale was used in the questionnaire. The term “never” received a score of 1, rarely: 2, sometimes: 3, often: 4, and always: 5. The minimum score of EMSRS was 31, and the maximum score was 155. Higher scores indicated more person's resilience. The validity of the questionnaire was confirmed in a study by Ebadi et al., and the reliability was confirmed using Cronbach's *α* coefficient (0.91) and the intrapolar correlation coefficient (0.85)^17^ (Appendix A1).

### 2.4 Procedures

Sampling was done in six phases. In the first phase, we visited the prehospital emergency department under the coverage of Qazvin University of Medical Sciences from April 8 to September 18, 2020. We explained the research method to all the prehospital emergency staff. Informed consent was obtained from the employees who met the inclusion criteria, and their contact numbers were recorded. An EMSRS was designed on WhatsApp and sent online to all operational staff of the centers on April 19, 2020. The participants were asked to complete the questionnaire within 48 h. Approximately 36 h after sending the questionnaire, a reminder text message was sent to those who did not complete the questionnaire. In addition, all those who had not completed the questionnaires by 48 h after sending the questionnaire were contacted via phone and asked to complete and send the online questionnaire.

To re‐evaluate the resilience of EMTs, the EMSRS questionnaire was administered to technicians on the 19th of May, January, July, August, and September through WhatsApp, and they completed the EMSRS questionnaire for 2 days. Follow‐up was performed as described in the first stage. Two hundred and thirty‐four individuals participated in the study for 6 months (April to September).

### 2.5 Sample size

Morgan's table was used to determine the sample size. At the time of sampling, approximately 351 people had worked in the prehospital emergency system of Qazvin Province, Iran. Therefore, the minimum sample size of this study was 183. However, to increase the credibility of the data and compensate for the possible loss of data, 30% was added to the sample size and 240 participants who met the inclusion criteria were included in the study using the census method. The response rate was 97.5%, and data from 234 prehospital emergency technicians were analyzed.

### 2.6 Statistical methods

All analyses were performed using SPSS‐16 software (SPSS Inc.). The Kolmogorov−Smirnov test showed that the data distribution was not normal. The data were analyzed using descriptive statistics, including frequency, mean, and standard deviation.

The Friedman test was used to demonstrate the relation between the EMS technician's EMSRS scores measured during 6 months. The Independent‐samples Kruskal−Wallis test was used to demonstrate the relationship between the participant's age, BMI, EMS work experience, number of shifts in the last months, and number of emergency missions per day with a mean of 6 months EMSRs. The Mann−Whitney *U* test demonstrated the relationship between the participants' marriage situation and EMSRS scores. The level of statistical significance was set at *p* < 0.05 for all statistical analyses.

## 3 RESULTS

**Figure 1:**
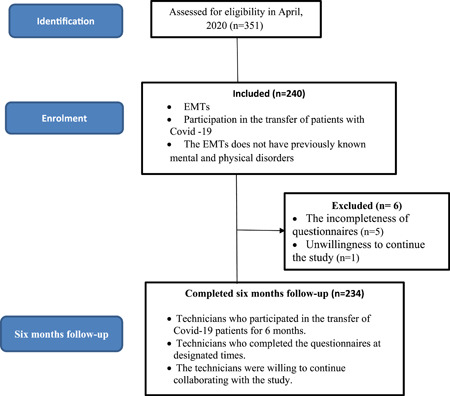
STROBE flow chart. EMT, emergency medical technicians.

Initially, 240 EMS technicians accepted to participate in the study, and 6 of them were excluded due to various reasons such as the incompleteness of questionnaires (5 subjects) and unwillingness to continue the study (1 subject). Therefore, the data of 234 EMS technicians were analyzed (Figure 1). The response rate was 97.5%.

All participants in the study were male because all emergency medical technicians are male in Qazvin providence. The mean age of the participants was 35.81 ± 15.97. The minimum and maximum ages of the participants were 21 and 56 years, respectively. The participants' BMI was within the range of 24−35, averaging 26.33 ± 4.27. Among the participants, 77.40% were married, and the rest were single. The EMS personnel's work experience was between 2 and 25 years, with an average of 10.55 ± 5.29. The participants' average number of shifts per month was 10.71 ± 1.92. The average number of Covid‐19 emergency missions was 11.71 ± 11.54 per/day during the study. There was no significant relationship between the mean of EMSRS scores in different age groups (*p* = 0.772), BMI categories (*p* = 0.840), work experiences categories (*p* = 0.356), and the average number of shifts per month categories (*p* = 0.942). There was a significant relationship between the mean of EMSRS scores and the average number of Covid‐19 emergency mission categories (*p* = 0.031) (Table 1).

**Table 1:** Participants' demographic variables and the relation of these variables with the average EMSRS scores measured during 6 months.

Variables	*n* (%)	EMSRS	Median	*p* Value^a^
		Mean ± SD		
Age (year)				
Young (18−35)	127 (54.3)	67.64 ± 7.31	35	0.772^a^
Middle‐aged (36−45)	91 (38.9)	66.97 ± 6.63		
Adult (46−65)	16 (6.8)	67.09 ± 7.10		
Marriage status				
Married	181 (77.4)	67.58 ± 7.03	‐‐‐	0.396^b^
Single	53 (22.6)	66.51 ± 6.98		
Number of children				
0	103 (44.0)	67.14 ± 7.35	1	0.389^a^
1	58 (24.8)	67.23 ± 7.41		
2	62 (26.5)	68.09 ± 6.27		
3	10 (4.3)	64.98 ± 6.00		
4	1 (0.4)	71.13 ± 0.00		
BMI				
Under‐weight (<18.5)	12 (5.1)	65.90 ± 8.41	35	0.840^a^
Normal (18.5−24.9)	44 (18.8)	67.71 ± 8.54		
Over‐weight (25−29.9)	178 (76.1)	67.34 ± 7.06		
Obese (>30)	0 (0)	0		
Working experience (year)				
<5	53 (22.6)	66.20 ± 7.01	11	0.326^a^
5−10	56 (23.9)	68.90 ± 7.97		
11−15	92 (39.4)	67.25 ± 6.59		
16−20	22 (9.4)	67.22 ± 6.64		
>20	11 (4.7)	65.85 ± 5.64		
Number of monthly shifts				
<5	3 (1.3)	66.6 ± 7.73	10	0.942^a^
5−10	128 (54.6)	67.52 ± 7.62		
11−15	101 (43.2)	67.17 ± 6.30		
>16	2 (0.9)	65.95 ± 1.11		
Number of Covid‐19 missions per day				
<5	103 (44)	68.24 ± 6.67	5	0.031^a^
6−10	47 (20.1)	67.41 ± 7.10		
11−15	27 (11.5)	67.68 ± 7.67		
>16	57 (24.4)	65.50 ± 7.04		

Abbreviations: BMI, body mass index; EMSRS, emergency medical services resilience scale.

^a^ Based on independent‐samples Kruskal−Wallis test.

^b^ Based on independent‐samples Mann−Whitney *U* test.

The mean scores of EMS employees' resilience were 66.74 ± 7.92, 67.53 ± 9.03, 67.52 ± 8.41, 67.32 ± 9.78, 67.01 ± 9.83, and 68.62 ± 6.98 from April to September, respectively (Table 2). The Friedman test indicated no significant difference between the mean scores of EMS employees' resilience during 6 months (*p* > 0.05). However, the correlation matrix between the scores of EMSRS during 6 months indicated that the resilience scores of EMS employees were positively correlated during the study (*p* < 0.01) (Table 3).

**Table 2:** Frequency distribution, mean, and relationship between EMS technicians' resilience scores during 6 months of study.

Months	EMSRS ranks	*n* (%)	Mean ± SD
April	Poor	68 (29.05)	66.05 ± 7.61
	Average	164 (70.08)	
	Good	2 (087)	
	Excellent	0 (0)	
May	Poor	69 (29.48)	67.48 ± 8.28
	Average	161 (68.82)	
	Good	4 (1.70)	
	Excellent	0 (0)	
June	Poor	60 (25.65)	67.00 ± 7.91
	Average	170 (72.65)	
	Good	4 (1.70)	
	Excellent	0 (0)	
July	Poor	56 (23.93)	67.08 ± 9.55
	Average	171 (73.07)	
	Good	7 (3.00)	
	Excellent	0 (0)	
August	Poor	51 (21.79)	67.24 ± 9.16
	Average	173 (73.94)	
	Good	10 (4.27)	
	Excellent	0 (0)	
September	Poor	35 (14.96)	68.08 ± 7.52
	Average	189 (80.77)	
	Good	10 (4.27)	
	Excellent	0 (0)	
Average of all months	Poor	57 (24.36)	67.34 ± 7.02
	Average	171 (73.08)	
	Good	6 (2.56)	
	Excellent	0 (0)	

Abbreviation: EMSRS, emergency medical services resilience scale.

*Based on Pearson's correlation coefficient.

**Table 3:** EMSRS scores correlation matrix during 6 months of study.

Months	April		May		June		July		August		September	
	*r*	*p*	*r*	*p*	*r*	*p*	*r*	*p*	*r*	*p*	*r*	*p*
April	1.000	0.000	0.610	0.000	0.553	0.000	0.544	0.000	0.533	0.000	0.508	0.000
May	0.610	0.000	1.000	0.000	0.716	0.000	0.738	0.000	0.722	0.000	0.645	0.000
June	0.553	0.000	0.716	0.000	1.000	0.000	0.779	0.000	0.714	0.000	0.672	0.000
July	0.554	0.000	0.738	0.000	0.779	0.000	1.000	0.000	0.798	0.000	0.883	0.000
August	0.533	0.000	0.722	0.000	0.714	0.000	0.798	0.000	1.000	0.000	0.747	0.000
September	0.508	0.000	0.645	0.000	0.672	0.000	0.883	0.000	0.747	0.000	1.000	0.000

Abbreviation: EMSRS, emergency medical services resilience scale.

The correlation matrix between the mean scores of EMSRS and demographic variables indicated that the scores of EMSRS had a negative correlation with the number of missions in the last 24 h (*p* = 0.026, *r* = −0.146). The job motivation subscale had a negative correlation with the number of missions in the last 24 h (*p* = 0.021, *r* = −0.151) (Table 4).

**Table 4:** Demographic variables and EMSRS subscale scores correlation matrix.

EMSRS subscales variables		EMSRS	Job motivation	Communication challenges	Social support	Remaining calm	Self‐management or self‐care	Consequences of stress
Age (year)	*r*	0.007	−0.084	0.044	0.103	0.010	−0.076	0.114
	*p*	0.910	0.201	0.501	0.116	0.879	0.249	0.083
MBI	*r*	−0.648	−0.548	−0.334	−0.420	−0.783	−0.564	−0.318
	*p*	0.164	0.260	0.518	0.407	0.065	0.244	0.539
Working experience (year)	*r*	0.046	−0.019	0.094	0.121	−0.007	−0.114	0.137
	*p*	0.488	0.775	0.152	0.064	0.912	0.083	0.037
Number of monthly shifts	*r*	−0.092	−0.124	−0.026	−0.009	−0.015	0.019	−0.070
	*p*	0.160	0.058	0.696	0.893	0.824	0.774	0.287
Number of Covid‐19 missions per day	*r*	−0.146	−0.151	−0.116	−0.003	−0.051	−0.027	−0.152
	*p*	0.026	0.021	0.075	0.968	0.435	0.678	0.020

Abbreviation: EMSRS, emergency medical services resilience scale.

The “consequences of stress” subscale was positively correlated with work experience in the emergency department (*p* = 0.037, *r* = 0.137) and negatively correlated with the number of missions in the last 24 h (*p* = 0.020, *r* = −0.152). There was not any statistically significant relationship between other subscales and demographic variables (*p* > 0.05) (Table 4).

## 4 DISCUSSION

Studies have indicated that the rates of well‐being and resilience decreased in all people, especially health workers and environmental services workers, during the COVID‐19 crisis in the world.^10^, ^18^, ^19^, ^20^ The present cross‐sectional study examined the resilience of 234 EMS employees who were continuously involved in transferring patients with COVID‐19 from prehospital settings to hospital emergencies for 6 months (April to September 2020). During the Covid‐19 pandemic, Iran's prehospital emergency system, like other countries in the world, was in an emergency state, and there was insufficient time to train prehospital emergency workers and implement mental health protocols.

During the 6 months, most EMS employees (73.08%) had moderate resilience based on EMSRS, and the participants' mean resilience scores were in the range of 67.01−68.62. Jaeger et al. found that the mean score of the resilience of EMS employees during the COVID‐19 crisis was 32.27 ± 4.8, according to a 40‐point tool.^21^ Meese et al. (2021) found that the resilience scores of administrative and Nonclinical, advanced practice providers, clinical support staff, and nurses were 6.47, 6.87, 6.51, and 6.57, respectively, according to an 8‐point tool after the COVID‐19 crisis.^10^ Miller and Brown found that 7.9% of EMS personnel had low resilience, and the rest had normal or excessive coping during the COVID‐19 pandemic.^22^ The participants' levels of resilience were higher than EMS staff in the present study during the outbreak of COVID‐19. Factors such as the lack of human resources in a prehospital emergency, working in intensive shifts, continuous use of PPE, distance from family, concerns about getting COVID‐19, seeing the death and critical illness of patients with COVID‐19, insufficient access to medical equipment, specialized drugs, and vaccines brought about the lower resilience of EMS staff, and subsequently, increase in the mortality and disability of patients with COVID‐19 in Qazvin provinces.

The repeated measures ANOVA indicated a significant difference between the mean scores of resilience in EMS staff during 6 months of measurement. The finding indicated that the EMS staff's resilience rate was moderate during 6 months, and no effective measures were taken to increase their resilience, which could affect the EMS staff's mental health and efficiency. In this regard, Gerami Nejad et al. (2019) reported a significant inverse relationship between resilience and shift fatigue. The more resilience decreased, the more the staff felt tired in their work shifts, thereby reducing their efficiency.^23^ Abrishamkesh et al. also found a significant relationship between EMS employees' resilience andmental health, that is, the higher the resilience of prehospital emergency staff, the higher their mental health (*p* < 0.001).^24^

The correlation matrix between the scores of EMSRS over 6 months of the study indicated that EMS staff's resilience scores were positively correlated during 6 months, revealing that the resilience of the previous month was influential on the resilience of the following month. It was also indicated that increasing or decreasing resilience in a period could increase or decrease resilience at later times; hence, the degree of resilience did not change much during the study since the degree of resilience in EMS staff decreased at the beginning of the COVID‐19 crisis and no measure was taken to increase it. Therefore, taking effective measures is recommended to increase the resilience of EMS employees who have no excellent resilience. Stress management is a measure that can be taken to increase EMS employees' resilience. Froutan et al. (2017) investigated the effect of implementing a stress management program on anxiety and resilience in emergency medical personnel. They indicated that stress management training could be effective in reducing anxiety and increasing resilience in emergency medical personnel. Reducing job stress may result in better clinical services. Thus, effective strategies are required to reduce job stress and increase resilience in EMS staff.^25^ As part of mitigation strategies, as resources could be particularly scarce during a serious pandemic situation, timely psychological support could also take many forms, including telemedicine and informal support groups.^26^ Kinlein and Karatsoreos showed in a study that stress, primarily processed via the hypothalamic‐pituitary‐adrenal (HPA) axis, engages biological pathways throughout the brain and body which promote adaptation and survival to changing environmental demands. Adaptation and resilience to environmental challenges is compromised when these pathways are no longer functioning optimally. The physiological and behavioral mechanisms through which HPA axis function influences stress adaptation and resilience are not fully elucidated.^27^ According to Kinlein and Karatsoreos study, the human body should be able to adapt to stressful factors after some time. However, in the current study, the level of resilience of prehospital emergency workers was almost constant during the 6 months of the study, and these technicians could not adapt to the stress caused by Covid‐19. Therefore, it can be said that caring for patients with covid‐19 in a prehospital emergency setting can suppress EMTs' ability to adapt and resilience against stressful factors. Covid‐19 was surprising in almost all areas of the health system. The health system has failed to design and implement a plan to reduce the stress of its employees dealing with Covid‐19. It seems that other health workers, such as nurses, physicians, allied health workers, and environmental cleaning workers, have a situation similar to that of prehospital emergency workers in terms of resilience against Covid‐19. However, this is not certain, and there is a need for further research. The findings of this study suggest that programs should be designed and implemented to reduce stress and increase the resilience of prehospital emergency workers in the phases of disaster mitigation and preparedness.

The questionnaires were sent online to the participants and they were asked to complete the questionnaires on their own. However, some participants might not have completed the questionnaires on their own. It is also possible that the participants suffered from a self‐reporting bias. To reduce the possibility of self‐report bias, this study was designed online so that the participants could complete the questionnaires anonymously. Therefore, this study has some limitations.

## 5 CONCLUSION

The EMS technicians' resilience was almost constant and moderate during the 6 months of care for patients with COVID‐19 and their transfer to the hospital, indicating that the COVID‐19 crisis could suppress the EMT's ability to increase resilience. The finding also indicated that the prehospital emergency employees' health was also at risk even though they worked hard to save lives and improve their health during the COVID‐19 epidemic crisis. Despite the fact that prehospital emergency workers are very effective in most crises and bear considerable mental and physical stress. However, the results of the present study showed that no serious measures were taken to increase prehospital technicians' resilience during crises.

## AUTHOR CONTRIBUTIONS

**Abbasali Ebrahimian**: Conceptualization; methodology; project administration; supervision; validation; writing—original draft; writing—review and editing. **Asghar Keshavarz‐Tork**: Investigation; methodology; resources; writing—review and editing. **Yousof Akbari‐Shahrestanaki**: Data curation; resources; writing—review and editing. **Maedeh Tourdeh**: Conceptualization; methodology; writing—review and editing. **Ali Fakhr‐Movahedi**: Conceptualization; data curation; methodology; writing—original draft; writing—review and editing. All authors have read and approved the final version of the manuscript.

## CONFLICT OF INTEREST STATEMENT

The authors declare no conflict of interest.

## ETHICS STATEMENT

This study protocol was approved by the Ethics Committee of Semnan University of Medical Sciences (approval ID: IR.SEMUMS.REC.1399.003, approval date: 2019.12.17). They were coordinated with Qazvin University of Medical Sciences before sampling. The study objectives and procedure were also explained to the participants, and informed consent was obtained.

## TRANSPARENCY STATEMENT

The lead author Ali Fakhr‐Movahedi affirms that this manuscript is an honest, accurate, and transparent account of the study being reported; that no important aspects of the study have been omitted; and that any discrepancies from the study as planned (and, if relevant, registered) have been explained.

## Data Availability

The authors confirm that the data supporting the findings of this study are available within the article. The data that support the findings of this study are available on request from the corresponding author. The lead author Dr. Ali Fakhr‐Movahedi has full access to all of the data in this study and takes complete responsibility for the integrity of the data and the accuracy of the data analysis.

## 1

(Table A1)

**Table A1:** Emergency medical services resilience scale.

No	Questions	Always	Often	Sometime	Rarely	Never
1	I participate in missions without motivation.	□	□	□	□	□
2	I am biased toward my career.	□	□	□	□	□
3	I stay in the organization because I have no other choice.	□	□	□	□	□
4	I try to get the injured person quickly to a Medical center.	□	□	□	□	□
5	I do my best all along the mission to survive the injured person.	□	□	□	□	□
6	I trust in my judgments and decisions on the scene of the incident.	□	□	□	□	□
7	I am responsible for what will happen to the injured person.	□	□	□	□	□
8	I control the challenges in the scene of the incident.	□	□	□	□	□
9	I can properly resolve the problems caused by missions.	□	□	□	□	□
10	At the scene of the incident, I want everyone to keep peace.	□	□	□	□	□
11	In every situation, I will volunteer to save lives	□	□	□	□	□
12	In case of close proximity to the accident site, I am immediately notified to the unit of deployment.	□	□	□	□	□
13	I volunteer to provide clinical services in crises.	□	□	□	□	□
14	Rescuing the injured person motivates me.	□	□	□	□	□
15	The satisfaction of the injured person motivates me.	□	□	□	□	□
16	I am get motivated by participating in decision making.	□	□	□	□	□
17	After hard and difficult missions I use humor to smooth the situation.	□	□	□	□	□
18	I am sympathetic to my colleagues.	□	□	□	□	□
19	At the scene of the incident, the behavior of the injured person and his companions is not understandable to me.	□	□	□	□	□
20	At the scene of the incident with I will cooperate with my colleagues, firefighting personnel and police in a professional manner.	□	□	□	□	□
21	I am nervous and hurried at the time of delivery of the injured person to the hospital emergency staff.	□	□	□	□	□
22	I enter into the scene of incident with faith in God.	□	□	□	□	□
23	In the face of accidents, I will pray.	□	□	□	□	□
24	My colleagues support me.	□	□	□	□	□
25	My colleagues advise me on solving the problems arise from missions.	□	□	□	□	□
26	Having past experiences has made me cope with the scene of incidents.	□	□	□	□	□
27	Before arriving at the scene I will make a mental image of the scene of the incident.	□	□	□	□	□
28	I will evaluate the safety and security of the scene before entering the scene.	□	□	□	□	□
29	I see the nightmare of my missions.	□	□	□	□	□
30	I have trouble sleeping.	□	□	□	□	□
31	I use medication to control my stress.	□	□	□	□	□

Ebadi et al.^17^
